# Vitamin D_2_-Enriched Button Mushroom (*Agaricus bisporus*) Improves Memory in Both Wild Type and APPswe/PS1dE9 Transgenic Mice

**DOI:** 10.1371/journal.pone.0076362

**Published:** 2013-10-18

**Authors:** Louise Bennett, Cindy Kersaitis, Stuart Lance Macaulay, Gerald Münch, Garry Niedermayer, Julie Nigro, Matthew Payne, Paul Sheean, Pascal Vallotton, Dimitrios Zabaras, Michael Bird

**Affiliations:** 1 Commonwealth Scientific and Industrial Research Organisation Preventative Health Flagship, Animal, Food and Health Sciences, Werribee, Victoria, Australia; 2 University of Western Sydney, School of Medicine, Campbelltown, New South Wales, Australia; 3 Commonwealth Scientific and Industrial Research Organisation Preventative Health Flagship, Materials Science and Engineering, Parkville, Victoria, Australia; 4 Commonwealth Scientific and Industrial Research Organisation Mathematics and Information Sciences, North Ryde, New South Wales, Australia; 5 Commonwealth Scientific and Industrial Research Organisation Animal, Food and Health Sciences, North Ryde, New South Wales, Australia; 6 Molecular Medicine Research Group, University of Western Sydney, Campbelltown, New South Wales, Australia; 7 Centre for Complementary Medicine Research, University of Western Sydney, Campbelltown, New South Wales, Australia; Cedars-Sinai Medical Center, Maxine-Dunitz Neurosurgical Institute, United States of America

## Abstract

Vitamin D deficiency is widespread, affecting over 30% of adult Australians, and increasing up to 80% for at-risk groups including the elderly (age>65). The role for Vitamin D in development of the central nervous system is supported by the association between Vitamin D deficiency and incidence of neurological and psychiatric disorders including Alzheimer’s disease (AD). A reported positive relationship between Vitamin D status and cognitive performance suggests that restoring Vitamin D status might provide a cognitive benefit to those with Vitamin D deficiency. Mushrooms are a rich source of ergosterol, which can be converted to Vitamin D_2_ by treatment with UV light, presenting a new and convenient dietary source of Vitamin D_2_. We hypothesised that Vitamin D_2_-enriched mushrooms (VDM) could prevent the cognitive and pathological abnormalities associated with dementia. Two month old wild type (B6C3) and AD transgenic (APP_Swe_/PS1dE9) mice were fed a diet either deficient in Vitamin D_2_ or a diet which was supplemented with VDM, containing 1±0.2 µg/kg (∼54 IU/kg) vitamin D_2_, for 7 months. Effects of the dietary intervention on memory were assessed pre- and post-feeding. Brain sections were evaluated for amyloid β (Aβ) plaque loads and inflammation biomarkers using immuno-histochemical methods. Plasma vitamin D metabolites, Aβ40, Aβ42, calcium, protein and cholesterol were measured using biochemical assays. Compared with mice on the control diet, VDM-fed wild type and AD transgenic mice displayed improved learning and memory, had significantly reduced amyloid plaque load and glial fibrillary acidic protein, and elevated interleukin-10 in the brain. The results suggest that VDM might provide a dietary source of Vitamin D_2_ and other bioactives for preventing memory-impairment in dementia. This study supports the need for a randomised clinical trial to determine whether or not VDM consumption can benefit cognitive performance in the wider population.

## Introduction

In 2010, dementia affected approximately 36 million people worldwide and is predicted to increase to 115 million by 2050 [1]. Alzheimer’s disease (AD) is the major contributor to dementia and the incidence is expected to rise 8-fold faster than the increase in population, with major implications for social and health care costs. The need to develop preventative and therapeutic interventions is urgent.

Whilst it is appreciated that dietary interventions such as reducing excessive fat, salt and sugar are beneficial for reducing the incidence of chronic diseases including cancer, diabetes and cardiovascular disease, few intervention studies have succeeded in linking diet to reducing the onset and symptoms of dementia, including AD. A recent report from the Australian Imaging, Biomarkers and Lifestyle Study of Ageing, showed that there was a significantly higher extent of adherence to the Mediterranean diet for healthy controls compared with either mild cognitively impaired or AD groups [2]. In addition there is growing evidence for beneficial effects of the micro-nutrient cocktail supplement on memory in AD patients Souvenaid [3], [4] and significant lowering of amyloid load has been demonstrated in animal dietary intervention studies with grape seed extract [5], curcumin [6], [7], resveratrol [8] and fish oil [9].

Vitamin D has also been proposed to affect central nervous system development, as supported by the association between its deficiency and incidence of some neurological and psychiatric disorders [10]. Furthermore, Vitamin D intake and metabolite status have each been positively correlated with cognitive performance [11], [12]. An observational study involving 225 aged individuals with probable AD exhibited Mini Mental State Examination scores that were positively correlated with serum 25-hydroxyvitamin D_3_ but not vitamins B1, B6 or B12 [13]. While this study did not demonstrate causality or mechanism, a functional role for Vitamin D in brain function has been implicated by comparative Single Nucleotide Polymorphism mapping of Vitamin D receptor (VDR) between AD and control patients and identified 2 sites: one with significantly increased risk and one conferring protection [14]. In addition, mRNA levels of the VDR and Ca-binding protein (calbindin) were significantly and selectively down-regulated in cells of the hippocampal region and not in the temporal cortex or cerebellum, in AD versus control patients [15]. Other combinations of polymorphisms of the VDR have also been associated with risk of late onset AD [16]. In addition, primary cortical neurons in the presence of Vitamin D were protected from amyloid beta peptide (Aβ1-42) toxicity by up-regulating VDR and suppressing apoptosis [17] and Vitamin D also protected SH-SY5Y cells against Aβ1-42 toxicity [18]. Finally, elevated expression levels of Vitamin D receptor and 24-hydroylase mRNA in rat hippocampal versus cortical neurons [19] also supports that Vitamin D is particularly important for calcium metabolism and function of hippocampal neurons and supports evidence that localised impairment of Vitamin D uptake in the hippocampus, which is affected in early stages of AD, can account for loss of cellular function including memory in AD patients.

Vitamin D directly or indirectly regulates more than 200 genes. All tissues and cells, including brain [20], contain a receptor for Vitamin D (Vitamin D receptor, VDR) that recognises the active form: 1, 25-hydroxyvitamin D [21]. Vitamin D exerts multiple bioactive roles, as a hormone, an anti-inflammatory agent and the regulation of cell growth. As the main route for obtaining vitamin D is through sun exposure, deficiency is prevalent in those with inadequate environmental exposure, which can occur for multiple reasons. For example, the elderly are at increased risk of vitamin D deficiency due to changed lifestyles associated with infirmity and loss of mobility. Vitamin D deficiency is also widespread in those at-risk of particular diseases, and is a risk factor for multiple chronic diseases [22].

Vitamin D supplementation benefits multiple aspects of health such as those at risk of falls and fractures (vitamin D is critical for Ca and bone homeostasis and Ca absorption), and lowers risk of cardiovascular, auto-immune diseases (eg, multiple sclerosis, arthritis), cancer [23] and Type 2 diabetes [22]. Vitamin D is reported to provide some protection against cardiovascular disease, specifically hypertension and cardiovascular mortality, and colorectal cancer with weaker evidence regarding immune-modulatory or anti-inflammatory effects [24]. However, apart from bone health, for which a ‘healthy’ Vitamin D metabolite blood plasma status is cited as >80 nM [25], the optimal metabolite levels of Vitamin D required for protection against specific disease states are not yet defined [26].

Consumption of edible mushrooms is considered good for health in general, with particular types having long histories of medicinal use in Eastern cultures. For example, treatment of mild cognitively impaired senior Japanese men and women with Yamabushitake mushroom (*Hericium Erinaceus*) was reported to improve cognitive function, that reverted after cessation of treatment [27]. This suggested that bioactives in these mushrooms other than Vitamin D were responsible for the effect and raise the possibility that Vitamin D-enriched mushrooms might act synergistically with other components.

The prevalence of Vitamin D-deficiency in both adults consuming the Mediterranean diet [28], and elderly populations dwelling in Mediterranean nursing-homes [29], highlights that dietary sources of Vitamin D are limited even in a ‘healthy’ diet. Vitamin D_2_ synthesis can be stimulated in mushrooms by UV irradiation [30] to produce a bioavailable dietary source of Vitamin D_2_ [31], [32], with capacity to promote Ca absorption and increase bone mineral density in mice [33]. The elevation of Vitamin D_2_ in mushrooms, in combination with other putative benefits for cognition, renders Vitamin D-enriched mushroom an interesting target for testing efficacy on brain function. A recent study of Vitamin D_3_ supplementation in Alzheimer’s disease model mice [34] demonstrated benefits of Vitamin D_3_ *per se* but the effects of the combination of Vitamin D_2_ and additional putative bioactives in mushroom, are not yet known.

This study aimed to determine the effects of dietary supplementation with Vitamin D_2_-enriched Button mushroom (VDM) in both wild type (B6C3) and a transgenic mouse model of familial AD (APP_Swe_/PS1dE9), compared with a Vitamin D-deficient base diet. Supplementation of VDM dried solids at 5% of feed (w/w) was given from 2 to 9 month old mice and study endpoints included learning and memory, toxicity by liver function biomarker, brain amyloid beta (Aβ) and inflammation biomarkers. The results are interpreted in terms of effects on learning and memory in the absence and presence of amyloid pathology in wild type and transgenic mice, respectively.

## Results

### Characterisation of Vitamin D_2_-enriched Button Mushrooms and Mouse Feeds

Analysis of the VDM solids was conducted to assess components other than Vitamin D_2_ that were introduced into the VDM diet and consequent compositional differences between the VDM and control diets (Table 1). Proximate analysis of VDM solids yielded protein, lipid, ash and carbohydrate concentrations of 25.9, 2.9, 12.4 and 58.8%, respectively. The protein content was in good agreement with a reported value for fresh Button mushroom (2.09% on fresh weight basis, equivalent to 26.1% on a dry weight basis at 92% moisture [35]. Approximately 23 to 40% of nitrogen in mushrooms is known to be present as free amino acids [35]. Inclusion of dried mushroom solids at 5% (w/w) in the Vitamin D-mushroom (VDM) feed, compared with control feed, produced an increase in concentration of ash by 12.9%. This accounted for variations in several trace elements between VDM and control feeds, specifically, elevation of levels of Cu, K and Mo by 11.2%, 12.6% and 10.7%, respectively, and lowering of levels of Ca and Mn by 5.2% and 5.0%, respectively (Table 1).

**Table 1:** Composition* of Vitamin D mushroom (VDM) solids, base feed, Control and VDM feeds, formulated from either 5% Vitamin D mushroom solids and 95% base feed (w/w, as is), or 100% base feed, respectively.

Component	Unit	Vitamin D mushroom	Base Feed#	VDM Feed	Control Feed	Difference-%
Total solids	%	92.14	89.23			
Protein	%	25.94	20.00	18.59	17.85	4.0
Total lipid	**%**	2.90	8.50	7.34	7.58	−3.3
Ash	%	12.40	3.24	3.32	2.89	**12.9**
Carbohydrate	**%**	58.77	68.26	60.12	60.91	−1.3
Ca	mg/100 g	13.75	1200.00	1017.86	1070.76	−**5.2**
Cu	mg/100 g	3.41	1.00	1.00	0.89	**11.2**
Fe	mg/100 g	3.51	5.10	4.48	4.55	−1.5
K	mg/100 g	3349.00	890.00	908.73	794.15	**12.6**
Mg	mg/100 g	110.00	250.00	216.99	223.08	−2.8
Mn	mg/100 g	0.58	12.00	10.20	10.71	−**5.0**
Mo	mg/100 g	0.13	0.04	0.04	0.04	**10.7**
Na	mg/100 g	70.35	350.00	299.93	312.31	−4.1
P	mg/100 g	1080.00	960.00	863.53	856.61	0.8
Zn	mg/100 g	5.98	6.00	5.36	5.35	0.1
Ergosterol	µg/kg	8282.00	24.80	402.57	22.13	**94.5**
Cholesterol	µg/kg	ND#	797.60	676.11	711.70	−**5.3**
Lanosterol	µg/kg	19.30	11.00	10.21	9.82	3.9
Vitamin D_2_	µg/kg	29.20	ND	1.35	ND	**100.0**
Vitamin D_3_	µg/kg	ND	ND	ND	ND	0.0

Standardised differences between VDM and Control feeds are shown as a percent of total solids. The moisture content of VDM solids and base feeds are taken into account but the moisture content of VDM and Control feeds were assumed to be equivalent.

* Methods used for analysis of VDM solids are described in Methods. Unless otherwise stated, analytical specifications of base feed were cited from information provided by supplier.

# Standard base feed containing 0.05 µg/g (2 IU) Vit D_3_ was fed for the first 8 weeks.

ND = not detected.

VDM feed contained 1.35 µg/kg (54 IU/kg) of Vitamin D_2_. No Vitamin D_2_ was detected in the control feed and there was no Vitamin D_3_ detected in either VDM or control feeds. Ergosterol, cholesterol and lanosterol were detected in the base feed but only ergosterol and lanosterol were present in the VDM. This led to elevation of ergosterol and Vitamin D_2_ by 94.5% and 100% between VDM and control feeds, respectively, and lowering of cholesterol by 5.3% in control feed (Table 1). It is possible that ergosterol in the VDM feed was additionally converted to vitamin D_2_ by exposure to fluorescent lighting during the course of the study. However, even if the concentration presented Table 1 is an underestimate, the impact on the conclusions from this study are not affected, firstly, because the level of Vitamin D_2_ in the VDM feed was intended to be elevated compared with the control feed, and secondly, effects of the dietary intervention are interpreted in terms of differences in resultant plasma Vitamin D metabolite levels.

### Mouse Biometric Monitoring

**Figure 1:**
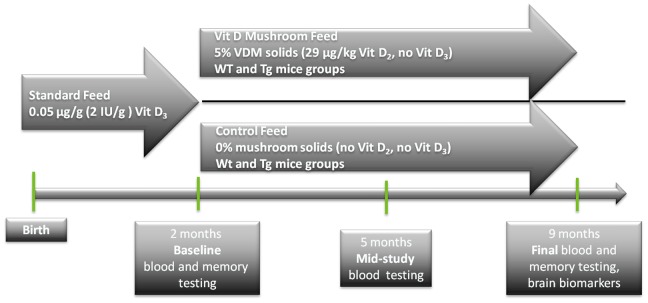
Schematic representation of the study design. Standard feed containing Vitamin D_3_ was fed for 2 months prior to baseline blood sampling and memory testing. Wild type and transgenic mice were then randomised into 2 groups and fed either Control or Vitamin D-loaded feeds for the intervention period of 7 months. The Control feed contained undetectable levels of Vitamin D_2_ and D_3_, as per Table 1.

The study design adopted for testing effects of VDM and Control feeds on Wild type and APPswe/PS1dE9 mice is presented in Figure 1. Mice of either genotype receiving either feed exhibited normal and equivalent rates of body weight gain over the trial period of ∼230 days. There was no significant effect of either genotype (WT versus Tg) or feeding treatment (VDM versus control) on overall weight gain over the 7 months of dietary intervention. Weight gains for each treatment group were as follows (in g): WT-VDM: 12.2±2.9; Tg-VDM: 13.6±5.1; WT-Control: 14.8±3.8 and Tg-Control: 14.4±6.3 (Table 2).

**Table 2:** Summary of differences in mouse body weight gain at 30 weeks of age, plasma protein levels at 6 months, plasma cholesterol and calcium at 9 months, for Wild Type (WT), transgenic (Tg) groups given either control or Vitamin D mushroom (VDM) feeds.

Measure	WT-control	WT-VDM	Tg-control	Tg-VDM
Body weight gain at 30weeks (mean, sd)	14.8±3.8	12.2±2.9	14.4±6.3	13.6±5.1
Total protein* (g/L, sem)	66.00±3.00	60.75±0.63	62.00±1.47	64.25±0.63
Albumin* (g/L, sem)	43.00±2.00	40.50±0.87	39.50±0.96	41.50±0.96
Globulin* (g/L, sem)	23.00±0	20.25±0.75	26.20±3.76	22.75±0.48
Total cholesterol (mM, sem)#	3.27±0.38	2.73±0.20	2.90±0.75	3.03±0.58
Ca (mM, sem)	2.51±0.03 (b)	2.42±0.02 (a)	2.60±0.03 (b,c)	2.49±0.03 (a)

* results represent the mean of n = 4 individual mouse sera.

# results represent the mean of n = 3 individual mouse sera.

a, b, c: different letter indicate significant differences (*P*<0.05).

### Liver Toxicity Biomarkers and Cholesterol in Plasma

Blood samples taken at the 6 month time point were analysed for selected liver toxicity biomarkers. Limited blood volumes prevented analysis of the complete set of liver enzymes. There was no difference either between genotype or feed for any plasma biomarker including: total protein, albumin and globulin (Table 2). These results suggest the absence of effects on liver function indicative of toxicity, with any genotype or treatment. Likewise, there was no change in plasma cholesterol either between genotypes or feeds, or within and between time points (Table 2).

### Calcium in Plasma

Plasma Ca levels were determined at the end of the study (9 months) and found to be significantly different between feeds with relatively higher levels measured in control (2.51 to 2.60 mM) versus VDM groups (2.42 to 2.49 mM, Table 2). The simplest explanation for the moderately but statistically significantly higher levels of serum calcium can be accounted for by the higher levels of dietary calcium in Control versus VDM feeds (by 5.2%, Table 1). The concentration of serum calcium of around 2.5 mM was comparable to the levels reported in mice fed a standard diet containing 1.1% Ca (1100 mg/100 g) [33] and the positive correlation between dietary and serum calcium reported in the same study can account for the slightly higher serum calcium in Control mice observed here. It is also possible that differences in plasma calcium levels also reflect differences in calcitonin and parathyroid hormone release in VDM versus Control fed mice, but influences of hormonal regulation cannot be confirmed from the available data.

### Vitamin D Metabolites in Plasma

After initial feeding with standard feed containing 0.05 µg/g (2 IU/g) Vitamin D_3_ and no Vitamin D_2_, mice received either VDM or Control feed containing 1.35 (54 IU/kg) or 0 µg/kg Vitamin D_2_, respectively, and neither VDM nor Control feed contained any Vitamin D_3_. The absence of Vitamin D_3_ in control and base feed was verified by multiple independent measurements. This feeding strategy tested effects of Vitamin D_2_ supplementation over a Vitamin D-depleted base diet in both WT and Tg mice. Additional putative bioactive species may be associated with the VDM solids however the study design did not permit the effects of Vitamin D_2_ from other mushroom bioactives to be resolved.

**Figure 2:**
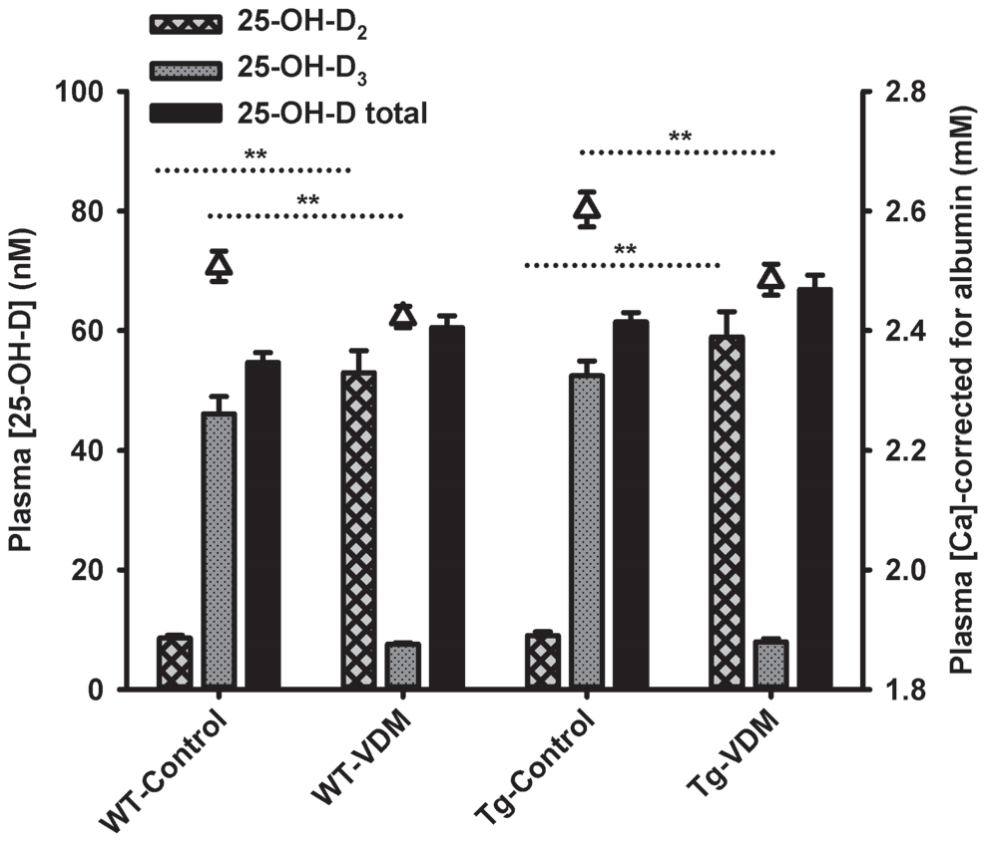
Effects of dietary interventions on concentrations of plasma metabolites of Vitamin D. Results are presented as a bar chart for 25-OH-D_2_, 25-OH-D_3_ and total 25-OH-D, overlaid with plasma levels of calcium (symbols), corrected for albumin, in bloods taken after 7 months of feeding experimental diets. Results represent the averages of duplicate analyses from at least 3 animals per group. Error bars represent standard errors of the mean and significant differences determined by 2-way ANOVA are shown (**, P<0.001). There was no difference between total 25-OH-D within genotype.

Levels of Vitamin D_2_ and D_3_ metabolites, 25-OH-D_2_ and 25-OH-D_3_, respectively, at the 9 month time point indicated highly significant differences in metabolite levels (Figure 2). While levels of total 25-OH-D of approximately 60 nM were equivalent across all treatment groups (P>0.05), levels of 25-OH-D_2_ and 25-OH-D_3_ displayed inverse ratios by a factor of approximately 5 in VDM and Control groups, respectively (Figure 2). Vitamin D adequacy was therefore achieved in all treatment groups and the effects of VDM versus Control feeding provided for a direct comparison between efficacy of Vitamin D_2_ (and mushroom solids) and Vitamin D_3_.

Total levels of the metabolite 25-OH-D ranged from 55 to 67 nM across the 4 groups of mice which was in good agreement with the range reported by Yu et al (2011) [34] for their transgenic and wild type mice fed from 2400 to 12,000 IU/kg of Vitamin D_3_, over 5 months, producing 50 and 110 nM of circulating 25-OH-D_3_ metabolite, respectively. The results indicated that intake of Vitamin D_2_ from mushrooms produced 25-OH-D_2_ as the predominant metabolite in both WT and Tg VDM groups, as expected. However, the Vitamin D_2_ intake in VDM groups appeared to suppress accumulation of Vitamin D_3_, known to have a slower turnover [36], as was observed in the control groups.

We propose that Vitamin D_3_ metabolite detected in the plasma of control-fed groups may have been produced from the conversion of tissue sources of pro-Vitamin D_3_ (7-dehydrocholesterol, 7-DHC) by mild UV exposure from fluorescent lighting in the mouse holding room, (M. Holick, personal communication). The observation that higher levels of dietary cholesterol in Control mice (by ∼5%, Table 1) did not produce higher levels of serum cholesterol (Table 2), might suggest that the serum cholesterol was partially utilised by Vitamin D-deficient Control mice. It is possible that the Vitamin D deficiency in the diet of Control animals stimulated gut enzyme-mediated conversion of cholesterol to 7-DHC [37], which was subsequently converted by mild UV exposure from fluorescent lighting to Vitamin D3 [38]. The positive correlation between serum cholesterol levels and capacity for UVB-mediated production of Vitamin D3 infers a biosynthetic relationship between circulating cholesterol, 7-DHC and Vitamin D synthesis [39], which is more efficient in circumstances of Vitamin D deficiency, ie, lower baseline levels of 25-OH-D metabolite produced higher levels of Vitamin D for the same quantum of UV exposure [39]. Thus, the utilisation of circulating cholesterol in combination with mild UV exposure can account for synthesis of Vitamin D3 in the Vitamin D-deficient Control mice.

### Learning and Memory Testing

Baseline evaluation of mice (at 2 months) in the Morris water maze (MWM) was compared for genotype groups (n = 24 mice per group) prior to commencing dietary intervention. There were no differences between genotypes in any training or probe testing parameters before commencing the dietary intervention (data not shown) indicating the absence of any pathology affecting memory in Tg mice at this time point.

**Figure 3:**
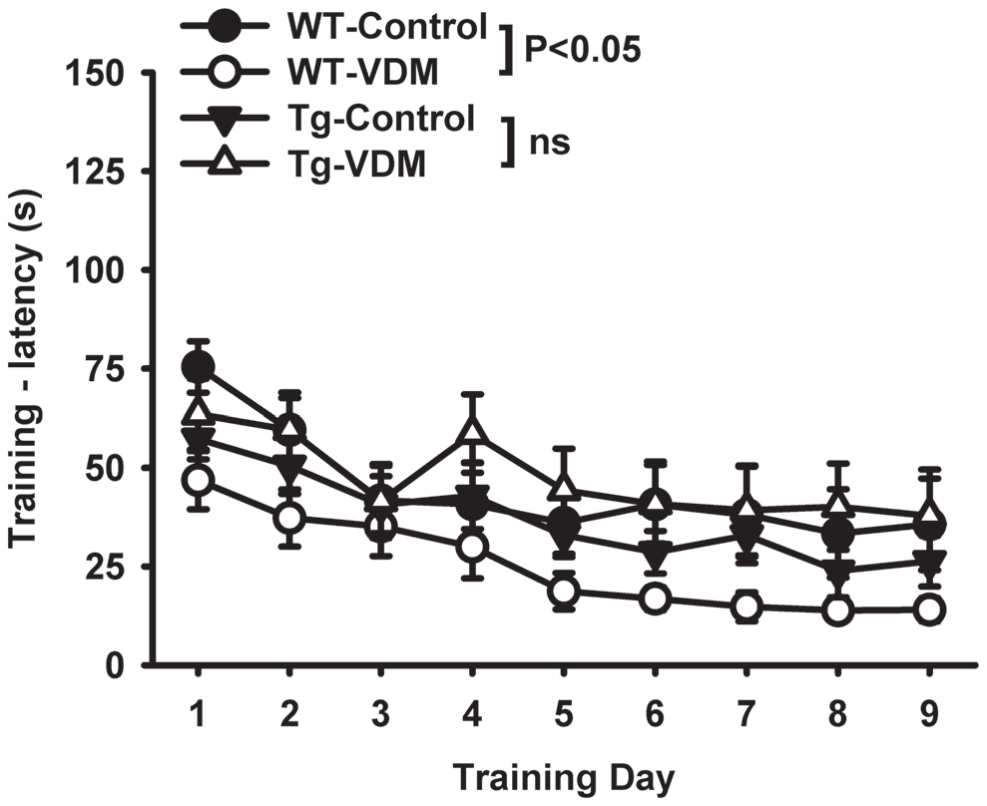
Dietary effects on Morris water maze training for wild type (WT) and transgenic (Tg) mice at 9 months. Results shown are latency to platform measure performed on 9 consecutive days. Results represent the average for the group with standard error at each time point. Learning rate for WT-VDM was significantly better than WT-control group by 2-way repeat measure ANOVA (feed, day) F (1,19) = 4.679, P<0.05).

The MWM testing on 9 months mice indicated that the rate of learning during training (latency to platform) was significantly faster (*P*<0.05) for WT-VDM compared with WT-control groups but there was no difference between Tg-control and Tg-VDM groups (Figure 3). There was no difference between any groups in the probe trial (data not shown).

**Figure 4:**
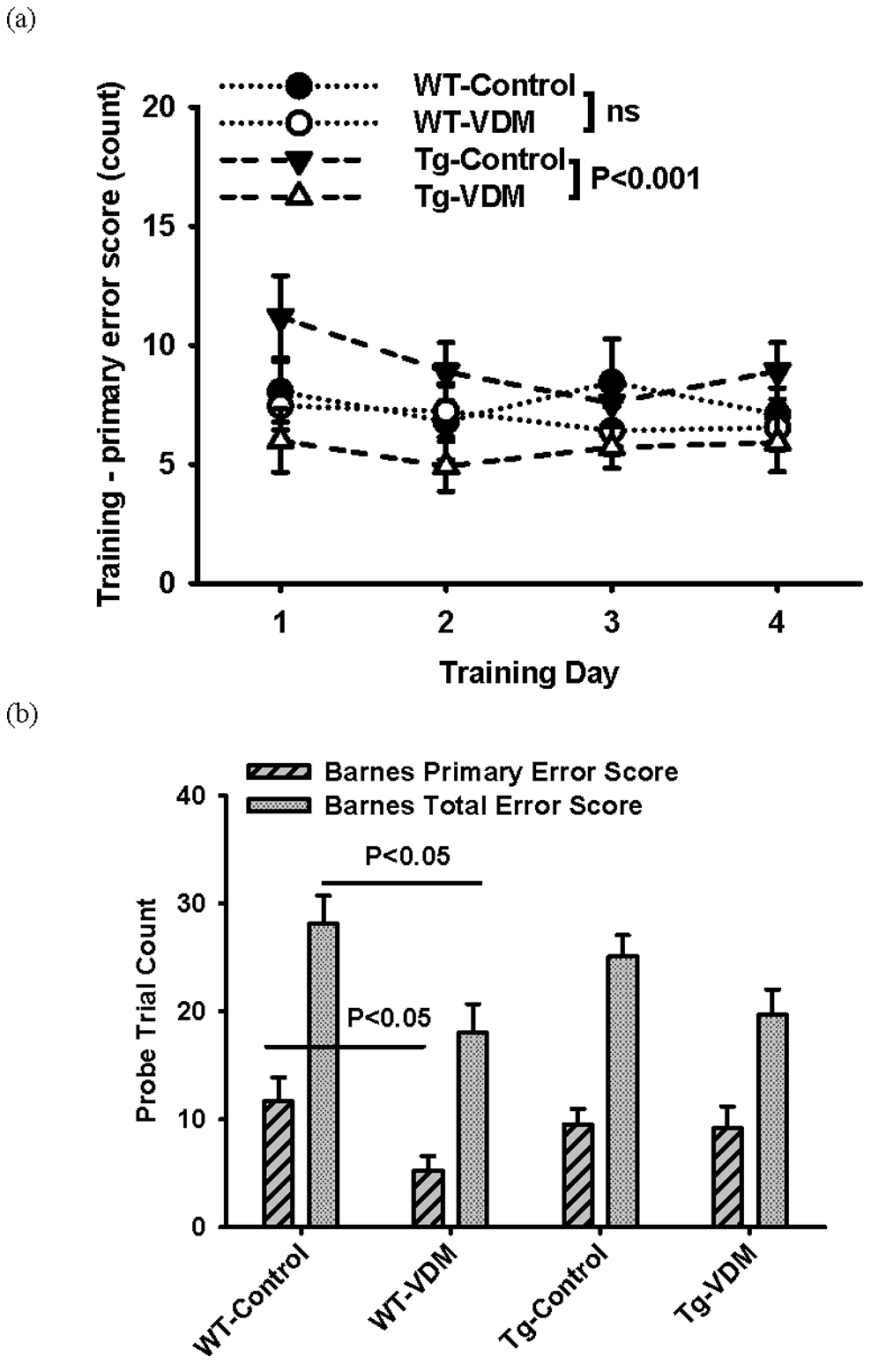
Dietary effects on Barnes maze training for wild type (WT) and transgenic (Tg) groups at 9 months. Results shown are (a) primary error score on 4 consecutive days, representing the average for the group and standard error at each time point. Tg-VDM were significantly more accurate than Tg-control group by 2-way repeat measure ANOVA (feed, day) F(1,23) = 13.092, *P*<0.001. (b) Subsequent probe trial testing on day 6 after removing escape hole showing primary and total error scores. Results represent the average for the group and standard error. For primary error score, the WT-VDM group was significantly more correct than the WT-control group by T-test (t = 2.186, P<0.05). For total error score, WT-VDM group was significantly more correct than WT-control group by T-test (t = 3.130, P<0.05).

Barnes maze testing, conducted on 9 month mice, revealed no effect of diet or genotype for any training parameters, except a diverging trend for distance and time travelled to find escape hole, favouring the VDM diet (data not shown), that was more apparent for Tg groups. There was significant benefit (*P*<0.001) of the VDM diet in Tg but not WT mice for the primary error score measure (Figure 4a). In contrast with MWM training where VDM consumption was more beneficial to WT than Tg mice (Figure 3), the training results for Barnes maze testing indicated a stronger benefit of VDM in Tg compared with WT mice. It is likely that the Tg mice experienced decreased physical robustness with age associated with disease progression and it is possible that the Barnes maze test was less physically demanding and stressful than the MWM, resulting in comparatively elevated cognitive performance in this setting.

The Barnes maze probe test showed that WT mice on the VDM feed had significantly (P<0.05) lower primary and total error scores, which was not significant for Tg groups (Figure 4b). There was no difference between diets for either genotype in measures of latency or distance travelled (data not shown). This may suggest that the delayed memory of Tg mice at 9 months was relatively more affected by the AD disease state, not present in WT mice.

**Figure 5:**
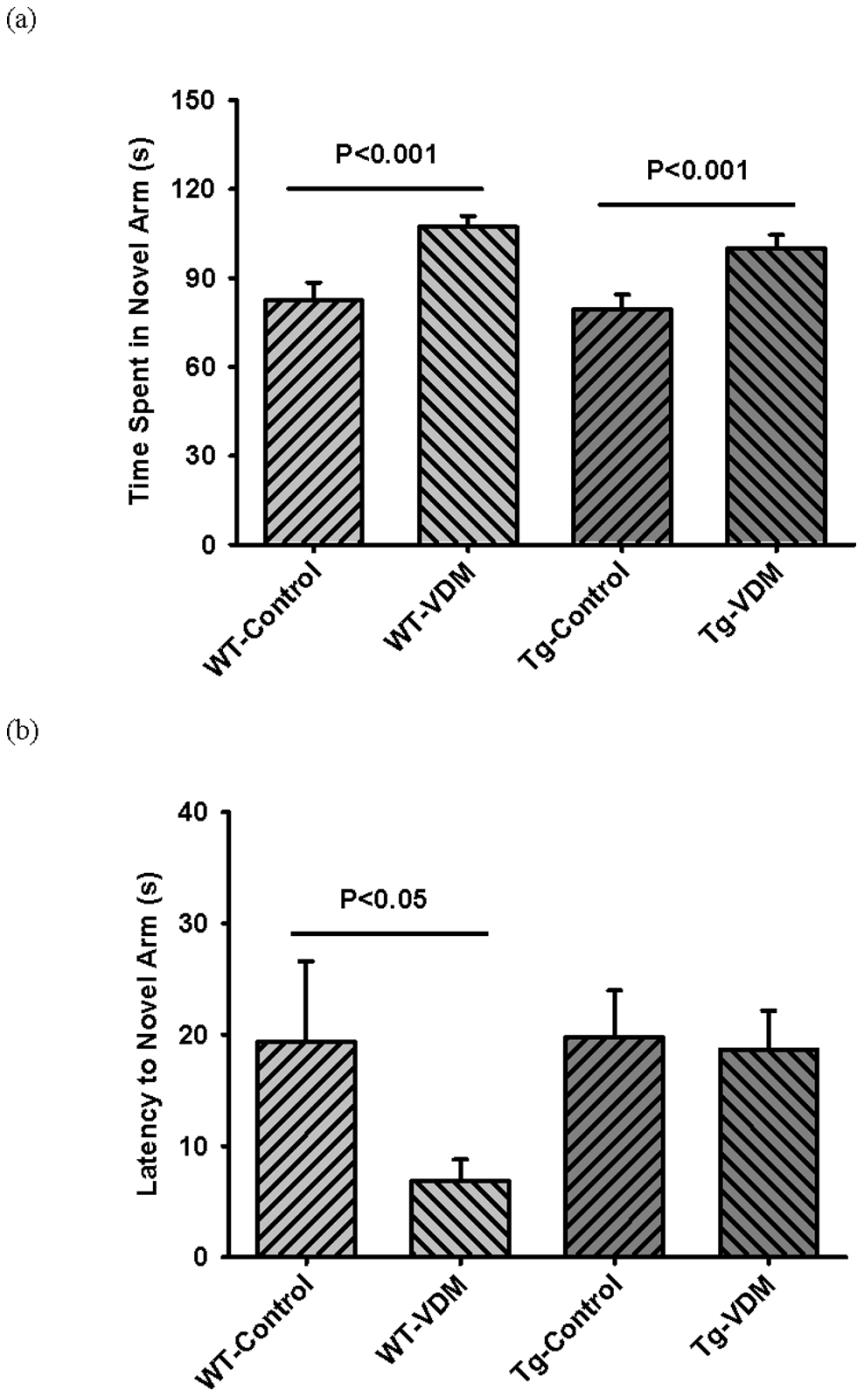
Dietary effects on Y maze probe trial at 9 months. Results shown are (a) time spent in novel arm and (b) latency to novel arm. Results represent the average for the group with standard error. For time in novel arm measure, WT-VDM stayed significantly longer than WT-control group by Kruskal-Wallis One Way ANOVA on Ranks (H = 23.731 with 3 degrees of freedom, P<0.001) whereas WT-controls spent similar time in all arms. Likewise, Tg-VDM group stayed significantly longer than Tg-control group by Kruskal-Wallis One Way ANOVA on Ranks (H = 21.324 with 3 degrees of freedom, P<0.001). WT-control and Tg-control groups spent similar times in all arms. For latency to novel arm measure, WT–VDM group showed improved learning by Mann-Whitney Rank Sum Test (U Statistic = 21.000, P<0.05).

Compared with mice on the control feed, both WT and Tg mice receiving the VDM diet spent significantly (*P*<0.001) more time in the novel arm of the Y maze (Figure 5a). The WT mice on the VDM diet were significantly (*P*<0.005) faster to enter the novel arm compared with WT mice on the control diet however, this effect was not observed in Tg mice (Figure 5b). In summary, significant benefits of VDM versus the control feed were observed in 3/5 memory tests for WT mice and 2/5 tests for Tg mice.

### Beta Amyloid in Plasma and Brain

**Figure 6:**
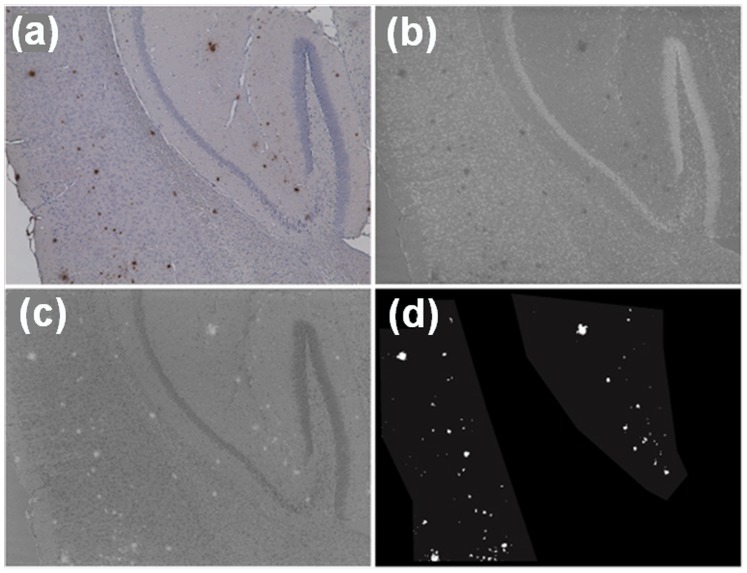
Visualising brain plaques using image segmentation methodology with Aβ42-specific 1E8 antibody. Original RGB image of typical transgenic brain section showing significant plaque presence (a) and nuclear image obtained using colour unmixing; plaques are mostly absent from this image (b). Plaque image obtained by colour unmixing; nuclei are mostly absent from this image while plaques are clearly visible (c). Masks for the cortex area and the hippocampus were produced manually as shown by lighter grey shading (d). Plaques shown in white were segmented automatically from image (c) using intensity Otsu thresholding on image (b). All images are 20×magnification.

**Figure 7:**
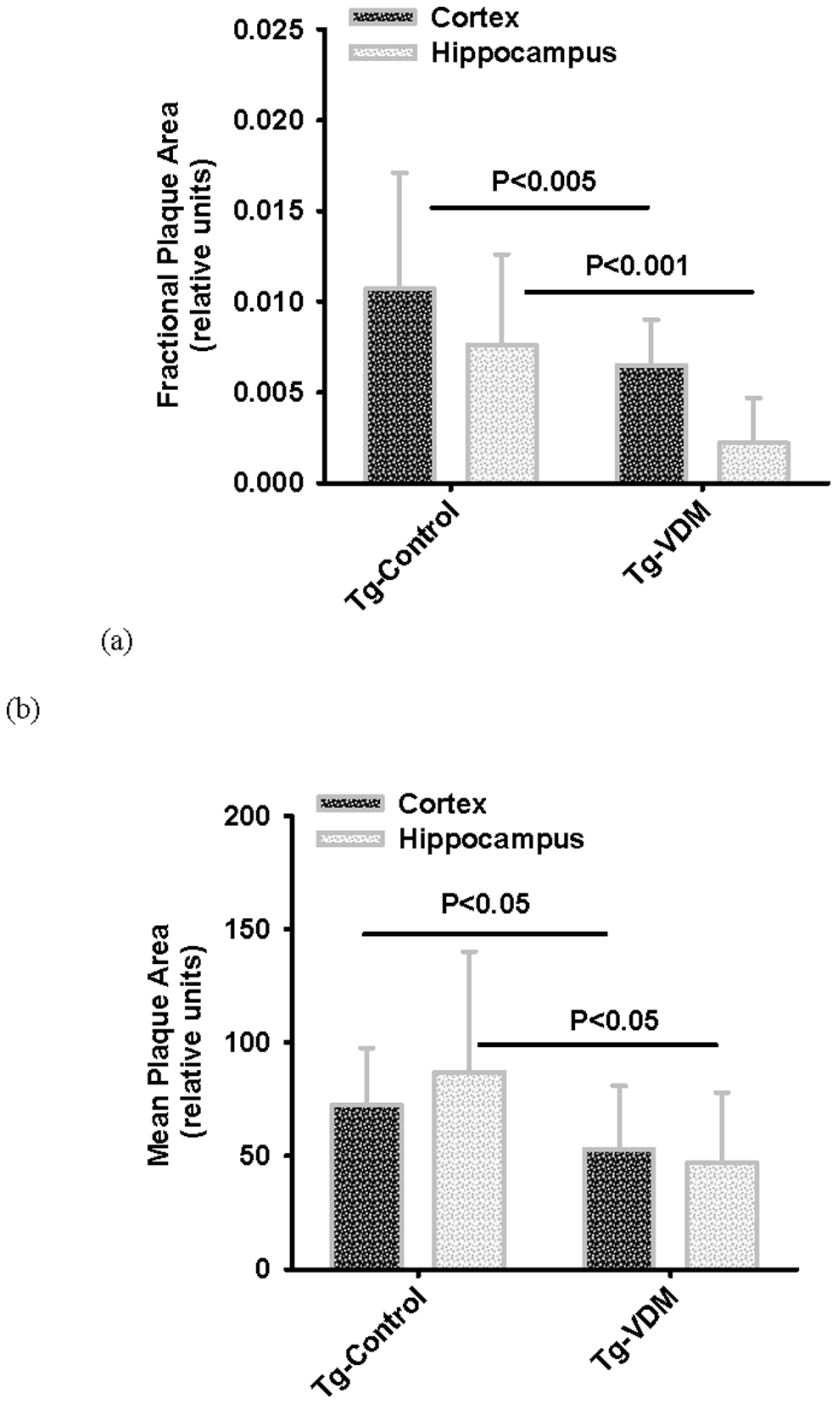
Quantification of brain amyloid plaque loads in transgenic mice groups at 9 months. Relative levels of brain plaque measured at 9 months, by immune-histochemical staining with 1E8 antibody to Aβ42 assessed by image analysis, showing (a) fractional coverage of plaque per total areas of cortex or hippocampus and (b) mean plaque areas in respective brain regions. Results are averaged across brains of n = 12 or 13 mice and are shown as average for the group with standard deviation. All *P* values were determined using Student’s T-test.

Plasma levels of both Aβ40 and Aβ42 in Tg mice increased significantly from 2 to 9 months however there was no difference between the diets at any time point (Table 3). The rise in level of plasma Aβ42, and presumably Aβ40, occurred between 2 and 6 months. Aβ plaque accumulation in the brain measured at 9 months by IHC with 1E8 antibody (Figure 6) indicated that compared with the control feed, Tg mice on the VDM feed had significantly less fractional Aβ plaque area per area of brain cortex or hippocampus (Figure 7a). In addition, the mean Aβ plaque size in the cortex and hippocampus was significantly smaller in the brains of mice on the VDM feed compared with the control feed (Figure 7b). However, without a measure of change in brain levels of amyloid plaque over time, it is not possible to speculate about relationships between serum and brain amyloid levels. The effects on plaque load suggested that either the VDM feed (either Vitamin D_2_ or mushroom bioactives) was effective in lowering Aβ42 expression, or alternatively that Vitamin D_3_ or deficiency of mushroom bioactives promoted Aβ42 expression in the Control group.

**Table 3:** Summary of plasma levels of Aβ40, Aβ42 measured at either 2 or 3 time points, respectively for transgenic (Tg) mice given either control or Vitamin D mushroom (VDM) feeds.

Measure	Time-mo	Tg-control	Tg-VDM
Aβ40 (pM)	2	441.4±33.7 (a)	463.2±40.8 (a)
	9	636.9±21.4 (b)	641.2±40.3 (b)
Aβ42 (pM)	2	165.4±10.6 (c)	153.6±12.2 (c)
	5	165.6±17.4 (c)	158.9±16.1 (c)
	9	197.0±9.1 (d)	185.0±17.2 (d)

Results are the mean and SEM of 7–12 mice per treatment group.

(a, b) Two-way repeat measure ANOVA showed significant difference from 2 to 9 months (*P*<0.05) for Aβ40 and for Aβ42, between 2 months and both 5 and 9 months (c, d, *P*<0.05).

### Inflammation in Brain

**Figure 8:**
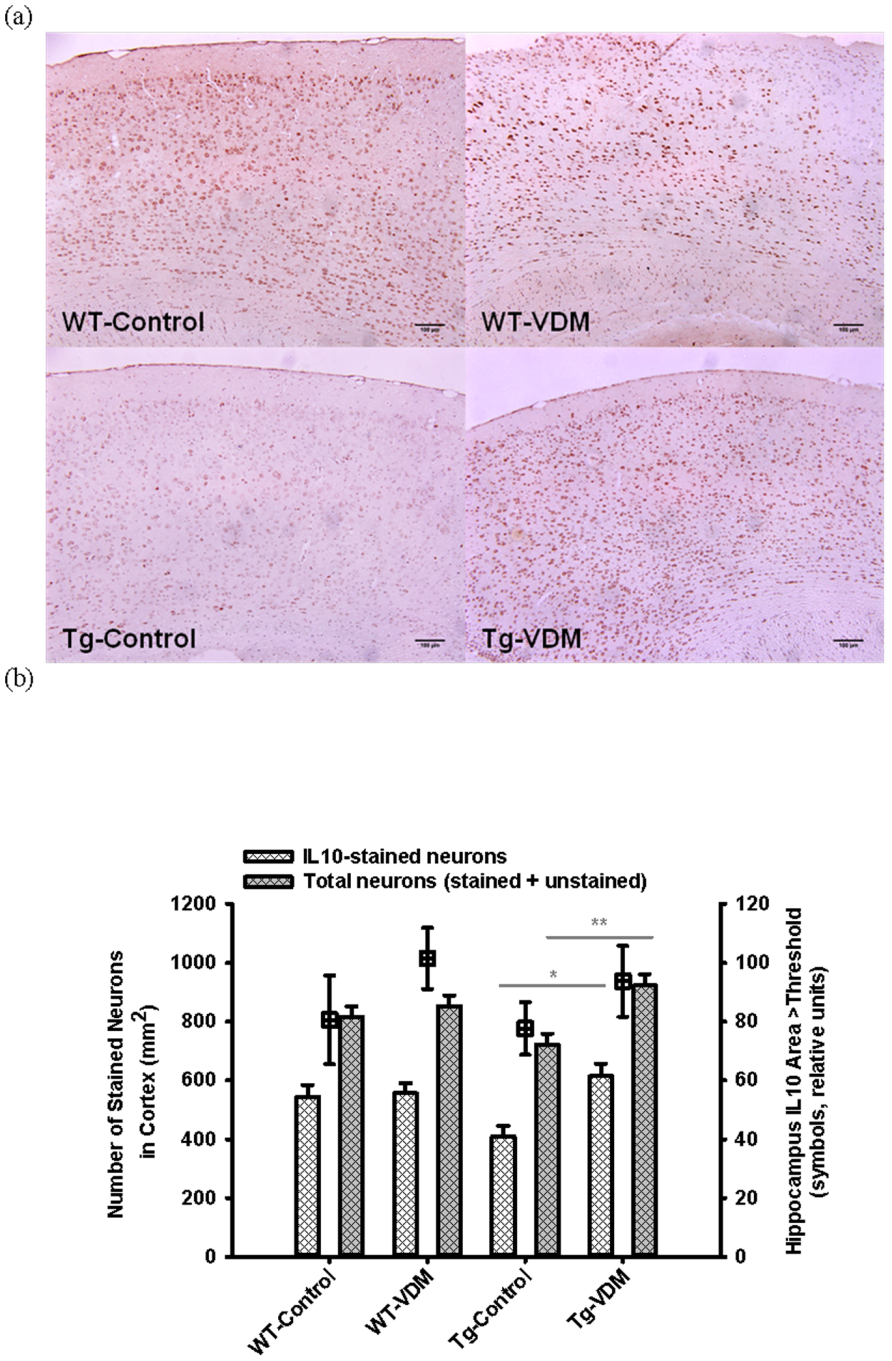
Immuno-histochemical quantification of IL-10-stained brain areas at 9 months. Typical images of IL-10-stained brains comparing mouse study groups (a) and results of quantitation by image analysis for the total cortex (neocortex plus temporal cortex, bars) and hippocampus (symbols) regions (b). Two way ANOVA analysis indicated significant effects of feed type (P<0.05) and interaction between genotype and feed type (P<0.05), with post-hoc feed type effect (Tukey test) significant for transgenic (IL-10- neuron area, P<0.001; total neuron number, P<0.05) but not wild type mice.

Compared with Tg mice on the control diet, Tg mice on the VDM diet had a significantly (*P*<0.05) higher number of IL-10-positive neurons in the cortex and a significantly (*P*<0.01) larger area of the neurons were IL-10 positive (Figure 8). A similar trend was apparent in the hippocampus, but was not statistically significant.

**Figure 9:**
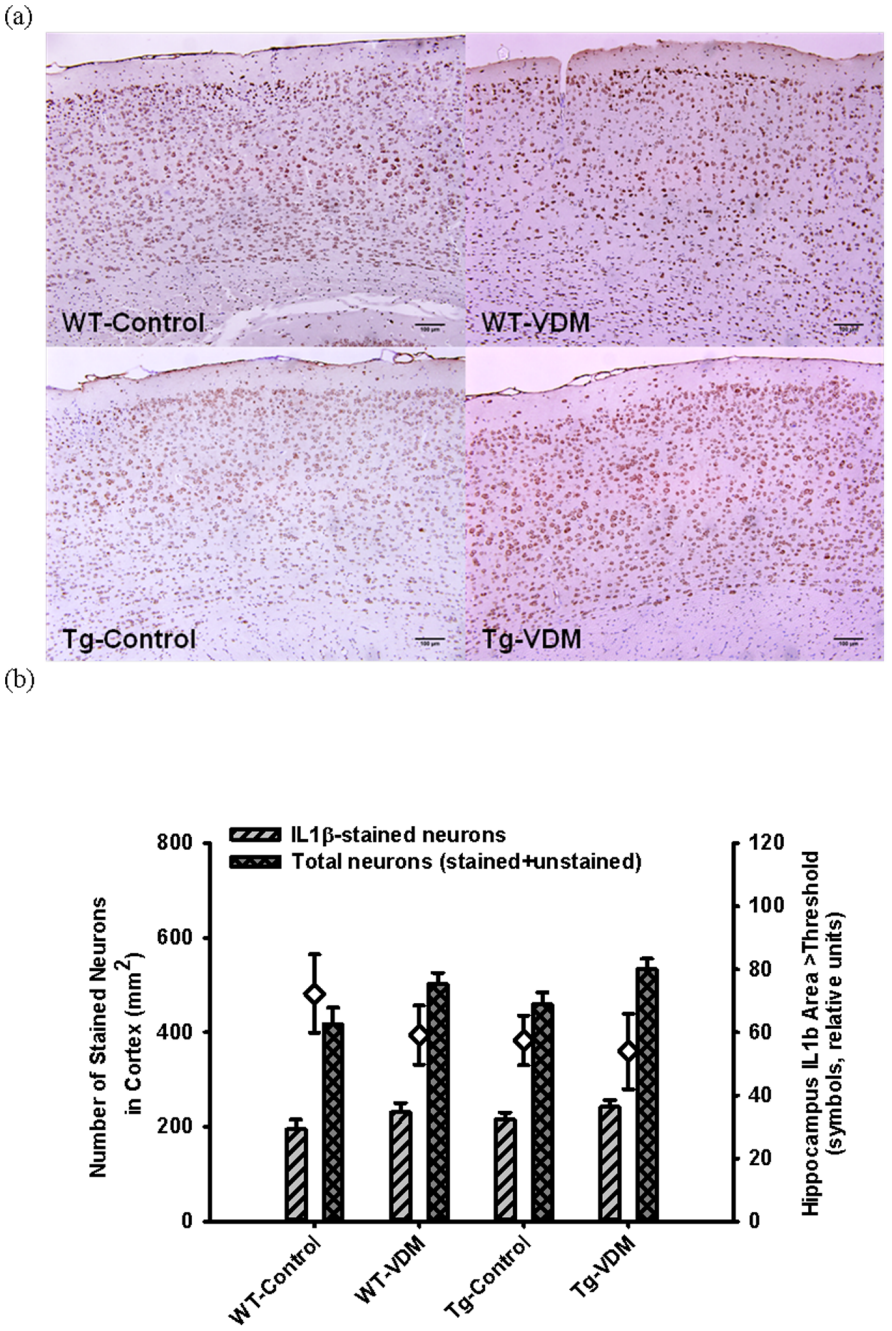
Immuno-histochemical quantification of IL-1β-stained brain areas at 9 months. Typical images of IL-1β-stained brains comparing mouse study groups (a) and results of quantitation by image analysis for the total cortex (neocortex plus temporal cortex, bars) and hippocampus (symbols) regions (b). Two way ANOVA analysis indicated no significant effects of either feed type or genotype on IL-1β-stained neuron area but a significant main effect of feed on total neuron number (P<0.05).

Immuno-localisation of IL-1β in the cortex or hippocampus of 9 months mouse brain sections showed no difference between feed type or genotype (Figure 9b) but a significant main effect of feed on cortex total neuron area,. The absolute sensitivity of IL-1β staining and magnitude of total neuron area (Figure 9) appeared to be lower than for IL-10 (Figure 8), based on comparison of stained areas.

**Figure 10:**
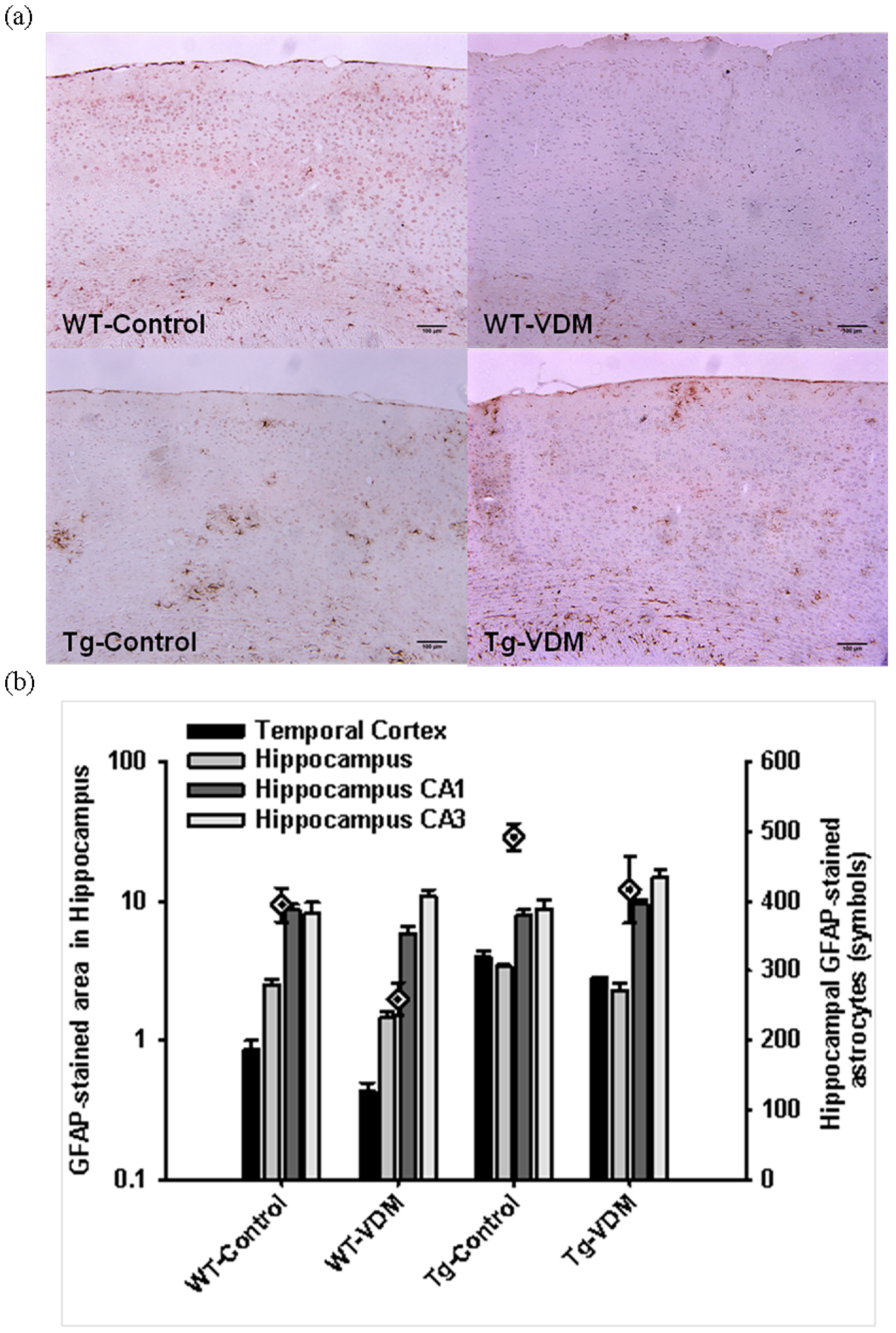
Immuno-histochemical quantification of GFAP-stained brain areas at 9 months. Typical images of GFAP-stained brains comparing mouse study groups (a), and results of quantitation by image analysis for the temporal cortex, total hippocampus, hippocampus regions CA1 and CA3 and hippocampal GFAP-stained astrocytes (symbols) (b). Two way ANOVA analysis indicated the following significant effects: genotype in the temporal cortex (P<0.001); both genotype and feed in the hippocampus (P<0.05); no effects in the CA1 hippocampus; feed (P<0.05) in the CA3 hippocampus; both genotype (P<0.05) and feed (P<0.05) for GFAP-stained astrocytes in the hippocampus. There were no significant interactions for any parameters.

Staining for GFAP measured at 9 months (Figure 10a) indicated a strong effect of genotype in the temporal cortex (P<0.001), a significant effect of both genotype and feed in the hippocampus (P<0.05), no effects in the CA1 hippocampus region and significant feed effect (P<0.05) in the CA3 hippocampus region. Significant lowering of GFAP-stained astrocytes in the hippocampus by VDM feeding (P<0.05) was evident for both genotypes (P<0.05), and supported that GFAP staining effects were specifically correlated with astrocytes in the hippocampus.

## Discussion

This study aimed to determine effects of a dietary form of Vitamin D, as present in UV light-treated mushrooms (Vitamin D_2_), together with other potential bioactive compounds, compared with a vitamin D-deficient control diet, on memory and cognitive performance in both a transgenic mouse model of AD and wild type mice. The vitamin D-deficient diet was introduced post-weaning, prior to which both the mothers and pups received sufficient vitamin D in their feed. As such, the study design modelled the scenario of adequate vitamin D status during early development followed by Vitamin D mushroom supplementation during juvenile and mature growth phases. This design thereby investigated possible benefits of Vitamin D supplementation (as VDM) to correct the decline in Vitamin D plasma metabolite status and prevalence of deficiency, that accompanies aging [40]. A weakness of the study design was the absence of treatment with non-irradiated (Vitamin D-free) mushroom, so as to separate effects of Vitamin D from non-Vitamin D mushroom components. As such, the result only permits comparison of the effects of the total Vitamin D-mushroom treatment versus the non-mushroom control diet.

Vitamin D status is positively associated with cognitive performance in both AD [41], [42] and non-AD subjects [43]–[47] suggesting that AD-independent and AD-specific effects of Vitamin D may apply. A non-causal relationship between vitamin D and AD has been reported [41], [48], [49], with evidence for its specific protective biological role also emerging [42], [50]–[52]. Vitamin D receptor (VDR) mRNA levels [53] are reduced in AD patients, and the gene encoding VDR has been highlighted as a risk factor in late-onset AD [14], [16], [54], [55]. By using WT and Tg AD mice, effects of dietary interventions in the absence and presence of AD pathology were compared and are discussed in terms of dietary effects common to both genotypes (genotype-independent), and those specific to the Tg AD genotype (genotype-dependent).

### Genotype Independent Effects - Nutrition

The elevation of Vitamin D_2_ levels in Button mushrooms by UV treatment [56] and its bioavailability to humans [57] has been established. At 5% (w/w) of the mouse base diet, VDM solids contributed a range of potential nutrients to the diet including Vitamin D_2_, other potentially bioactive molecular derivatives of ergosterol formed in proportions that depend on the UV wavelength profile [58], ergothioneine, a potent anti-oxidant [59], polyphenolics including hispidins [60] and selected minerals (Table 1).

Minerals with greater than 5% boost in the VDM feed included: Cu, K and Mo. Copper (Cu) is used by a range of metallo-enzymes including dopamine beta monoxygenase, which converts the neurotransmitter dopamine to norepinephrine, and superoxide dismutase, a cellular anti-oxidant. Potassium (K), with sodium, is important for maintaining electrolyte balance and function of nerve and muscle cells. Molybdenum (Mo) is also a co-factor for several metallo-enzymes involved with catabolism of sulphur amino acids and DNA bases [61].

Minerals that were diluted by more than 5% included: Ca and Mn. Calcium (Ca) is a key requirement for normal skeletal development, neuromuscular and cardiac function and likewise, manganese (Mn) is also essential for bone formation. In addition, Mn is required for several metallo-enzymes involved with metabolism of carbohydrates, cholesterol and amino acids [61] and is a co-factor for mitochondrial super-oxide dismutase (SOD).

These differences between Control and VDM feeds did not manifest in any detectable effects on mouse growth (Table 2), cholesterol or any biomarkers of toxicity (Table 2). However, it is worth noting that the Tg-VDM group only displayed statistically significant ‘slowness’ during behavioural testing at 9 months (data not shown) that might have reflected effects of Ca and Mn dietary depletion on skeletal development and possibly compromised mitochondrial SOD-mediated anti-oxidative capacity in the Tg-VDM mice. Therefore, only behavioural results that are independent of speed have been reported.

### Genotype Independent Effects – Vitamin D Status

The experiment presented here effectively compares low dose Vitamin D_2_ derived from fresh Button mushroom solids with low dose Vitamin D_3_ arising from environmental UV exposure in response to a Vitamin D-deficient diet in WT and Tg mice. The metabolite status results provide interesting insights into the competitive bioavailability of Vitamin D_2_ and D_3_, which has been reported in humans using Vitamin D supplements [36] and also for mushroom-derived Vitamin D_2_ [57], but not previously in mice. Indeed, Vitamin D metabolite status is commonly reported as total 25-OH-D, which does not take into account either the Vitamin D_2_ to D_3_ intake ratio or the active metabolite ratio. As observed in this study for mice, when Vitamin D_2_ was taken as a supplement over either summer or winter by senior women (∼70 years old), the metabolite status of 25-OH-D_2_ was inversely correlated with 25-OH-D_3_ [62]. Thus, when oral or environmental forms of Vitamin D_3_ are co-administered with Vitamin D_2_, the metabolism of Vitamin D_2_ appears to be strongly favoured over Vitamin D_3_. This effect is distinct from the reported higher efficiency of Vitamin D_3_ metabolism into 25-OH-D_3_ compared with Vitamin D2 metabolism into 25-OH-D_2_, when given separately [36].

The VDM groups tended to have slightly higher total levels of 25-OH-D (25-H-D_2_+25-OH-D_3_) compared with Control-fed groups (Figure 2), as a possible consequence of the cumulative contributions of both dietary Vitamin D_2_ and endogenous sources of Vitamin D_3_. However, this trend was not significant suggesting that the benefits of the VDM feed were related to either the superior effect of Vitamin D_2_ over Vitamin D_3_, or synergistic effects of Vitamin D_2_ with bioactive factors present in the VDM. Comparative safety and efficacy of Vitamin D_2_ and Vitamin D_3_ supplementation are in early stages of evaluation [36], stimulated by new dietary sources of Vitamin D_2_, such as mushrooms. It is clear that if efficacies of Vitamin D_2_ and Vitamin D_3_ on different health endpoints, ie, bone health cf. cancer cf. cognition, are found to diverge, these findings should inform future guidelines for Vitamin D supplementation and definitions of ‘healthy’ Vitamin D plasma metabolite status. Recently, all-cause mortality risk was reported to follow a ‘J’ curve that associated negative consequences for survival with levels of 25-OH-D >90 nM [63]. It is likely that the previous data was specific for 25-OH-D_3_, as Vitamin D_2_ is prescription-only in Denmark where the study was conducted (personal communication), but it is not yet known if the J-curve relationship and the optimal range of Vitamin D status would be the same for Vitamin D_2_ or combinations of Vitamin D_2_ and D_3_, as a result of increasing availability of dietary sources of Vitamin D_2_.

### Genotype Independent Effects – Learning and Memory

The APPswe/PSEN1 mouse is a well-characterised rodent model of familial AD, exhibiting both histological and behavioural hallmarks of the human condition. These AD mice exhibit learning deficits in the Morris water maze memory test [64]–[68], while spontaneous alternation behaviour and performance in the traditional Barnes maze appears to be intact [69], [70]. In the present study, we used a novel arm approach in the Y-maze and observed no difference between genotypes, complementing previous studies conducted using spontaneous alternation [69], [70]. APPswe/PSEN1 mice were not expected to display any impairments in motor co-ordination or general physical capacity compared with wild type littermates [70], and the AD genotype cannot explain the observed slowness of the Tg-VDM group in Morris water and Barnes maze tests, which has been attributed to possible dietary depletion of Ca and Mn (Table 2). Neither can loss of visual acuity and other deficits that have been reported to occur in older (20–26 months) APP\swe/PS1dE9 Tg mouse [71] account for slowness in these mice at 9 months of age.

This is the first study to report effects of mushroom-derived vitamin D_2_ on cognitive outcomes in both WT and AD Tg mice. The study design exploited competitive effects of Vitamin D metabolism and permitted a virtually direct comparison between efficacy of Vitamin D_2_ and Vitamin D_3_, based on Vitamin D metabolite levels (Figure 2). Learning and memory performance was consistently superior for VDM versus Control groups of both genotype, possibly implicating effects on cognition of Vitamin D_2_ to be superior to Vitamin D_3_. Reported benefits of Vitamin D_3_ supplementation on learning and memory in both APPswe/PSEN1 [34] and aged, male F344 rats [72] is also consistent with these findings.

The significance of Vitamin D adequacy for brain development and function is of growing concern in the context of neurodegeneration and aging. Neuroprotective effects are related to the action of Vitamin D_3_ on neurons and glial cells and roles in biosynthesis of neurotrophic factors, including glial-derived neurotrophic factor (GDNF) and nerve growth factor (NGF) [73], and neurotransmitters. Other important functions are the regulation of inflammation via the inducible nitric oxide synthase (iNOS) pathway, and astrocyte-mediated brain detoxification by elimination of reactive oxygen and nitrogen species involving gamma-glutamyltranspeptidase and glutathione [74]. Vitamin D_3_ controls NGF signalling with important effects on hippocampal neuron survival, neurite outgrowth and neurotrophic signalling [73], [75]. The *in vitro* and *in vivo* studies of Vitamin D effects on brain function have mostly been tested with Vitamin D_3_. Whether or not Vitamin D_2_ exerts comparable functions to Vitamin D_3_ is not known and the current study infers that Vitamin D_2_ may be more efficacious for neuroprotection.

Alternate interpretations of this data reflect the potential contribution of non-Vitamin D mushroom bioactive species, conferring either additive or synergistic contributions, to Vitamin D on improvement in learning and memory. Components of mushrooms other than Vitamin D_2_ may also provide for neuroprotection. A Malaysian mushroom extract of *Pleurotus giganteus* was shown to promote outgrowth of PC12 cells that was attributed to both secondary metabolites of the sterol and triterpene class and also to effects of K [76]. Although Button mushroom secondary metabolites have unknown bioavailability to brain, the high K associated with VDM feed (Table 2) may also account for neurotrophic effects in the mice brains leading to improved memory and learning in these groups.

### Genotype Dependent Effects – AD and Amyloid Pathology

APPswe/PSEN1 mice initially present with amyloid deposits at approximately 4 months of age, and amyloid deposition increases with disease progression [77]. Changes in levels of Aβ40 and Aβ42 in blood reflect changes in distribution between the brain and periphery over time. With disease progression, the ratio of Aβ42 to Aβ40 was higher in the brain compared with blood, measured at 6 and 12 months of age for APPswe/PSEN1 mice [77] and similarly for the Tg2576 model [78]. The Aβ peptide levels were accompanied by detectable increased memory deficit from 6 months of age [79]. The intervention with a VDM diet significantly lowered the brain load of Aβ42 (Figure 7) but peripheral levels of Aβ42 and Aβ40 were unchanged (Table 3). These data suggest that a VDM diet may significantly affect either the expression or processing of APP, favouring a non-amyloidogenic pathway, but not the efflux of the amyloidogenic products, Aβ42 or Aβ40.

These effects of VDM are in contrast to frequent reporting of *inverse* correlations between changes in brain and blood Aβ2 levels. For example, in the APPswe/PS1dE9 mouse model, an inverse relationship between brain deposits of Aβ42 and serum levels was induced by either vaccination-mediated production of anti-Aβ42 antibodies in the periphery [80] or a combination of anti-Aβ42 antibodies and active vaccination [81]. In addition, a new drug for treating vascular complications of diabetes also lowered brain Aβ42 while increasing plasma Aβ42 in APPswe transgenic mice [82]. These examples most likely reflect enhanced efflux of brain Aβ42 without affecting production rates of Aβ42 in the brain.

Mice on the VDM diet showed lower Aβ42 brain plaque load without change in plasma Aβ42 suggesting that Vitamin D_2_ (or mushroom bioactives) altered APP processing, as the expression rate of APP was expected to be strongly controlled by the transgenic genotype. Stimulation of non-amyloidogenic processing of APP by the VDM diet is supported by another study which showed that AD Tg mice treated with Vitamin D (12,000 IU/kg feed) had reduced Aβ42 peptide and plaque load in the brain and increased non-amyloidogenic peptide products of APP [34]. The effect was attributed to possible inhibition of beta secretase or enhanced clearance by NGF-stimulated astrocytes [34]. It is not known if Vitamin D_3_ therapy also lowered blood Aβ42 levels, which was not reported. In further support, we have shown that a beta secretase inhibitor factor was present in Wood Ear and may also be present in Button mushrooms [83]. Furthermore, neither Vitamin D_2_ nor D_3_ appear to interact directly with Aβ42, according to ThT-binding assay (data not shown), and is therefore unlikely to directly modulate brain clearance, but may do so by Vitamin D/VDR-mediated processes [17], [72].

### Genotype Dependent Effects – Inflammation

This study has demonstrated that, compated with control feed, VDM-fed Tg mice displayed higher numbers of IL-10-immunopositive cells (Figure 8), lower GFAP-stained astrocytes (Figure 10) in the temporal cortex and hippocampus and higher total neuron count (Figure 8). The elevation of total neuron area in the cortex by the VDM feed supported that mushroom bioactives were bioavailable to brain and were responsible for a neuroprotective effect. Effects on GFAP were also observed in VDM-fed WT mice (Figure 10). The results of this first study to quantify IL-10 and IL-1β in this mouse model using IHC, are in good agreement with ELISA-based methods [34], [72].

IL-10 is an anti-inflammatory, neurotrophic cytokine produced by microglial and neuronal cells within the CNS [84], [85]. IL-10 inhibits cytokine production within monocytes and macrophages [86], [87] having a suppressive effect on the inflammatory cascade. The elevation of total neuron count due to IL-10-expressing neurons observed in VDM-fed Tg mice (Figure 8b) suggested that the VDM feed promoted an anti-inflammatory response. No effect of VDM was observed in WT mice suggesting that the inflammation was associated with amyloid plaque deposition. Amyloid pathology did not appear to influence the primary pro-inflammatory cytokine, IL-1β.

The Tg and WT mice on the VDM diet had lower numbers of GFAP-positive astrocytes compared with mice on the Control diet. GFAP-positive astrocytes were shown to accumulate near amyloid plaque [88] and VDM-mediated effects can therefore be explained by the overall lowering of plaque area and size by VDM feeding (Figure 7). In a similar AD Tg mouse model and in the absence of any intervention, hippocampal GFAP was unchanged between 5 and 14 months for WT mice but increased significantly by 14 months for Tg mice [88]. This suggested that the inflammatory response in AD Tg mice occurs at later stages and as a consequence of plaque deposition [88]. Our data agrees with the previous study as we did not observe a change in GFAP-positive cells in the hippocampus of 9 month Tg compared with WT mice on the Control diet. In contrast, the Tg mice on the VDM diet showed significant lowering of GFAP-stained area in the cortex and hippocampus, compared with Tg mice on the Control diet. The GFAP-stained area tended to increase in the hippocampus CA1 and CA3 regions, with VDM-feeding in the Tg compared with the Control-fed mice. These data show that there is an overall benefit of VDM feeding, but important brain region-dependent effects.

VDM feeding in Tg mice resulted in a significant increase in total neuron numbers compared with Tg-Control groups (Figure 8b). Trophic effects of vitamin D have been linked with stimulation of Nerve Growth Factor (NGF) in Tg AD mouse brain [34] and the inhibition of nitric oxide [89]. The current data does not permit confirmation of neurotrophic effects of VDM by NGF but is supported by these reported *in vivo* effects of vitamin D and also by stimulation of neuronal growth by mushroom-derived compounds *in vitro* [76], suggesting that the bioactive combination in VDM may exert synergistic effects on neuronal growth.

The attribution of the collective effects of the VDM feed to Vitamin D *per se* (i.e., not other mushroom bioactives) is supported by the comparable effects of Vitamin D_3_ supplementation in AD Tg mice, where up-regulation of NGF and down-regulation of TNFα were observed. Likewise, beneficial regulation of inflammation by subcutaneous injection of Vitamin D_3_, specifically, up-regulation of IL-10 and down-regulation of IL-1β, was reported in aged F344 rats, devoid of amyloid pathology [72]. If these effects in either Tg or non-Tg mice are specific to Vitamin D, results from the current study might suggest that Vitamin D_2_ (VDM feed) is more efficacious than Vitamin D_3_.

Through the suppression of amyloid deposition and consequent inflammatory immune responses, Vitamin D ameliorates cognitive decline in the AD mouse model. Considering the presence of Vitamin D receptors on neurons and glia [90], together with accumulating epidemiological evidence [91], [92], these data support the role of Vitamin D in the modulation of inflammation in either the absence [72] or presence of AD pathology [34] and benefits for cognition.

Collectively, these results justify further investigation of the usefulness of Vitamin D-enriched Button mushroom as a protective dietary factor against AD in randomised clinical trials. Further studies are necessary to resolve possible differential efficacies of Vitamin D_2_ versus D_3_ on cognition, and specific effects of non-Vitamin D components of mushroom.

## Materials and Methods

White button mushrooms (Sylvan 737 strain) were obtained from Adelaide Mushrooms (Montaro, South Australia, Australia). Ergosterol (vitamin D_2_), cholecalciferol (vitamin D_3_), L-ergothioneneine, lanosterol and 7-dehydrocholesterol were purchased from Sigma Aldrich (St Louis, MO, USA) and vitamin D_2_-[^2^H_3_] standard in ethanol (Isosciences, King of Prussia, PA, USA).

### Preparation of Vitamin D-enriched Mushrooms

Mushrooms were washed, air dried and chopped into halves prior to UV treatment. UV treatment to drive the conversion of ergosterol to Vitamin D_2_ was conducted using a light-proof box (1300 mm width×1300 mm depth×500 mm height) assembled with an array of 6 parallel UV-C tubes (Philips TUV 36W.G36 T8 Longlife UV-C lamps, North Ryde, NSW, Australia), as described in Liu et al, 2009 [93]. After 2 hr stabilisation time, the UV-C light intensity was 1700 µW/cm2 measured using a radiometer with a solar blind photodiode SED 240 sensor and cosine-correction diffuser (Model IL1700, International Light, MA, USA), set at 253.7 nm. The intensity was up to 30% lower at the edges compared with the centre and also lower because the treatment plane of the mushrooms was 30 cm further away from the UV source compared with the radiometer. Mushrooms were treated in 2 batches: 25 and 5 kg treated for 12 and 30 s, respectively, before freeze-drying. The batches of dried mushrooms were vacuum-sealed in sub-samples and stored at 4°C, and blended as required at a ratio of 2∶1 to yield an average Vitamin D_2_ concentration of 30 µg/kg.

### Proximate Analysis

The dried mushroom product was analysed for total moisture using a HR73 Halogen moisture analyser (Mettler Toledo, Port Melbourne, Victoria, Australia). Total nitrogen analysis was conducted by the flash combustion method using a Carlo Erba Elemental Analyser (Model 1108, Carla-Erba, Milan, Italy), with a coefficient of variation for replicates of <2%. Total lipid analysis was determined gravimetrically after Mojonnier extraction [94]. Total ash analysis was conducted by gravimetric analysis of residue after thermal oxidation of sample in a platinum crucible before drying to constant weight in a dessicator. Carbohydrate content was calculated by difference. The protein level was computed from the N content using the recommended factor of 4.55 for Button mushroom [35].

### Mineral Analysis

The dried mushroom product was digested by microwave digestion in a mixture of concentrated nitric and hydrochloric acids (80∶20, v/v) according to the US EPA Method 3051 (1994). Analysis of digestates was conducted by Inductively-Coupled Plasma Atomic Emission Spectroscopy (ICP-AES) on a Varian Vista Pro instrument (Varian Australia, Melbourne, Australia) under optimised settings, in at least duplicate, with appropriate reagent blanks and reference samples. The average coefficient of variation between replicates was 10%.

### Analysis of Vitamin D and Related Species

Vitamin D_2_ (ergosterol), vitamin D_3_ (cholecalciferol), lanosterol and 7-dehydrocholesterol stock solutions were prepared by dissolving in ethanol. The concentration of vitamin D_2_ was confirmed using ε at 265 nm of 18,843 cm-1 [95]. A solution of [^2^H_3_]-vitamin D_2_ (20 µg/ml) in ethanol was used as an internal standard.

Extraction of Vitamin D and related compounds was adapted from Japelt et al. [95]. Replicates of freeze dried samples (100 mg) were weighed into 25 ml glass tubes with Teflon lids and mixed with 60% (w/v) potassium hydroxide (1 ml); 96% ethanol (v/v, 5 ml), 15% (w/w) ascorbic acid (3 ml) and [^2^H_3_]-vitamin D_2_ internal standard (20 µl). Samples were agitated at 22°C for 18 h before adding 20% ethyl acetate in pentane (7.5 ml) and mixing for a further 30 min. Sample tubes were centrifuged at 2000×g for 5 min at 22°C and the organic layer recovered. Extraction into ethyl acetate was repeated and the combined extracts were then washed with 50 mM HCl (10 ml) and the complete removal of alkali confirmed by pH testing. The organic layer was transferred to a round bottom flask and dried by rotary evaporation at 30°C. The residue was redissolved in 1% iso-propanol in n-heptane (2.5 ml) before loading onto a silica cartridge column (500 g resin, 4 mL reservoir), previously activated with n-heptane (5 ml). After washing with 0.5% (v/v) iso-propanol in n-heptane (2×5 ml), bound compounds were eluted under vacuum with 6% (v/v) isopropanol in n-heptane (2×4 ml) and the eluant evaporated to dryness under nitrogen. The residue was finally redissolved in 100% methanol (3 ml) and filtered (0.2 µm) before LC-MS/MS analysis.

For LC-MS/MS analysis, the sample (3 µl) was injected and separated on a C18 column (XTerra MS, (2.1×150 mm, 3.5 µm particle size, Waters Corp., Milford, MA, USA) at 30°C with isocratic elution (0.5% (v/v) formic acid in methanol at a flow rate of 200 µl/min over 15 min). The detector was a Quantum triple stage quadrapole (TSQ) mass spectrometer (Thermo Fisher Scientific, Scoresby, Vic, Australia) equipped with an atmospheric pressure chemical ionisation (APCI) source. Samples were scanned in positive mode with selected reaction monitoring (SRM) of products identified by the following m/z ions and retention times: Vitamin D_3_, 259.2, 5.64 min; Vitamin D_2_ and [^2^H_3_]-vitamin D_2_, 159.1, 5.1 min; 7-dehydrocholesterol, 159.1, 5.9 min; ergosterol, 159.1, 6.04 min; lanosterol, 191.1, 7.11 min and cholesterol, 161.1, 7.12 min.

### Preparation of Experimental Mouse Feeds

Mouse feed pellets were prepared by crushing the vitamin D_3_-depleted base feed (custom-made product, Barastoc mice cubes, Ridley Agriproducts Pty Ltd, Victoria, Australia; proximate and micronutrient analysis given in Table 1) milled to particles of <2 mm. Vitamin D-rich mushroom powder was dry-blended with crushed mouse feed at 5% (w/w) to a final Vitamin D concentration of ∼1±0.2 µg/kg, before dispersing in de-ionised water to form a moist dough. Control feed was prepared in the same manner but without addition of mushroom solids. After thorough mixing, the dough was manually-fed through a mincer with a sausage attachment (Weston No. 8 Manual Meat Grinder, Pragotrade USA) to make pellets of approximately 4 cm length and 1.5 cm diameter. The pellets were oven-dried overnight at 60°C before vacuum sealing and storing at 4°C until use. Feeds were prepared in batches of 1–3 kg, as required. Assuming an average daily consumption of 3 to 5 g per mouse, the daily dose of Vitamin D_2_ was 3 to 5 ng (0.12 to 0.2 IU).

### Mouse Feeding Study

Transgenic (Tg) mice expressing human genes for the Swedish variant of amyloid precursor protein and exon-9 deleted presenilin-1 (APPswe/PS1dE9) were obtained from the Jackson Laboratory (Stock 004462; Bar Harbour, ME, USA). Experimental subjects (all males) were selected from a breeding colony of hemi-zygous male crossed with wild type (WT) female mice. Genotype identification was performed prior to experiments by the PCR protocol recommended by the Jackson Laboratory. All experiments were performed in accordance with the Prevention of Cruelty to Animals Act, 1986 under the guidelines of the National Health and Medical Research Council (NHMRC) Code of Practice for the Care and Use of Animals for Experimental Purposes in Australia, and approved by the Animal Ethics Committee (AEC) of the Howard Florey Institute, University of Melbourne (AEC No. 11-010).

A total of 21 WT and 25 Tg mice completed the study, which were randomised between four groups based on genotype and feed type: *n* = 10 wild type, control diet (WT-control); *n* = 11 wild type, Vitamin D mushroom diet (WT-VDM); *n* = 13 transgenic, control diet (Tg-control) and *n* = 12 transgenic, Vitamin D mushroom diet (Tg-VDM). Standard mouse feed (Vitamin D_3_-replete, Barastoc, Ridley Agriproducts Pty Ltd, Victoria, Australia) was fed to all mice up to completion of the baseline Morris water maze testing at 2 months of age, after which VDM and control feeds were substituted and fed for a further 7 months (Figure 1, Table 1). Mice were housed in treatment groups (3–5 per cage) in an artificially lit environment with 12 h light/dark cycles and no exposure to natural light. Experimental feeds and water were available *ad libitum* for the duration of the study. Monitoring for weight and health was performed twice per week and behavioural testing was done at the same time point in the light cycle.

### Blood Sampling

Blood samples were taken from mice at baseline (2 months), middle (6 months) and end time points (9 months). At 2 and 6 months, samples were collected via sub-mandibular bleeds using Goldenrod animal lancets (MEDIpoint Inc., Mineola, NY) in K3 EDTA tubes (Greiner Bio-One, Kremsmunster, Austria, item no. 450475). At the conclusion of behavioural testing (9 months), blood samples were taken prior to perfusion with 4% paraformaldehyde. Mice were anaesthetised (80 mg/kg sodium pentobarbitone, i.p.) and blood collected via cardiac puncture. Blood was divided between EDTA tubes (for Aβ42 analysis) and lithium-heparin tubes (Greiner Bio-One, item no. 450477) for Vitamin D analysis. The tubes were mixed by inversion 5–10 times before centrifuging at 2000×g for 10 min. The upper plasma layer was removed by pipette, dispensed into 1.5 ml centrifuge tubes and snap-frozen in liquid nitrogen prior to storage at −80°C until use.

### Plasma Protein Analyses

Protein levels were determined as an index of liver function using EDTA tubes, using Cobas colorimetric auto-analyser methods for Total Protein and Albumin (Gen.2, Roche Diagnostics, IN, USA), and calculating globulin by difference. Plasma from 4 animals per treatment group was tested.

Cholesterol was determined using the Cobas colorimetric auto-analyser method for cholesterol (Gen.2, Roche Diagnostics). Plasma from 4 animals per treatment group 2, 6 and 9 month time points were tested.

Calcium was determined using the Cobas colorimetric auto-analyser methods for Calcium and Albumin (Roche Diagnostics).and corrected for albumin-bound Ca. Plasma from 4 animals per treatment group at the 9 month time point, were tested.

Analysis of Vitamin D metabolites was determined using plasma from 3 animals per treatment group at the 9 month time point. Analysis of metabolites 25-hydroxyvitamin D_2_ and D_3_ was conducted using liquid chromatography with tandem mass spectrometry as described previously [96].

Analysis of Aβ1-40 and Aβ1-42 was conducted using commercial ELISA kits for detecting human β-Amyloid peptides (Wako, Richmond, VA, USA), according to the manufacturer’s instructions. Transgenic mouse plasma samples were diluted by a factor of 16 and analysed as independent duplicates by comparison with a standard curve. Plasma from at least 7 animals per treatment group at either 2 and 9 month time points (Aβ40) or 2, 6 and 9 month time points (Aβ42) were tested and the results reported as the average and standard error of the mean.

### Behaviour Testing by Morris Water Maze

Prior to introducing test feeds (∼2 months of age) and at the conclusion of the feeding study (∼9 months of age), hippocampal-dependent, long term spatial learning and memory retention were assessed in wild type and APPswe/PS1dE9 mice using a Morris water maze [97], [98]. Ethovision XT tracking software (Noldus, Leesburg, VA, USA) was used for data collection, and water temperature was maintained at 25±3°C. Subjects were required to utilise external spatial cues in order to locate the platform. During memory training, mice underwent four trials per day with approximately 15 min between each trial. Entry point into the maze was randomised. Mice that were unable to complete the task within 2 min were placed on the platform for 20 s before removal from the maze. After each trial, mice were dried and placed under a heat lamp to prevent hypothermia. Acquisition of spatial memory was assessed by examining latency and distance travelled by the mouse to locate the platform, for 9 consecutive days of training. On Day 10, a “probe” trial was performed to test memory retention and retrieval. The probe trial involved the removal of the platform and the subject was monitored for the length of time spent in the “home” quadrant.

### Behaviour Testing by Barnes Maze

Long-term, hippocampal-dependent, spatial reference memory was also assessed using a 36 hole Barnes maze (diameter = 120 cm) at the 9 month time point [99]. In order to increase motivation, mice were exposed to a loud buzzer and aversive bright lights during each trial, both of which were switched off upon entering the target hole. Mice were introduced into the maze from underneath a small box which was remotely raised by the experimenter in the adjoining room via a pulley system. After a pre-trial in which mice were led by the experimenter to the target hole, mice were exposed to the maze twice per day, with a 30 min break, for four consecutive days. Parameters measured were primary latency (time until first visit of target hole), latency to enter target hole, primary error score, number of errors and total error score (for the purposes of error scoring, each hole was designated a number between 0 [target hole] and 18 [opposite to target] according to distance from the target). Trials were a maximum of 2 min long, and were terminated prior if the mouse was successful in entering the escape hole. A probe trial was conducted on Day 5, where the escape box was removed and the latency and number of visits to the target hole determined.

### Behaviour Testing by Y-maze

A standard Y-maze was utilised in order to assess short-term spatial memory [100], [101]. The maze comprised three enclosed arms (length = 30 cm, width = 10 cm, height = 16.5 cm), each with a distinct visual cue located at the end, as well as a centre zone located at the point at which the arms met. Protocol was performed as previously described [102], [103]. Briefly, mice were initially exposed to two of the three arms for 10 min, and were then removed to home cages for 2 h. Mice were subsequently re-exposed to all three arms of the Y-maze (including the novel arm) for 5 min. Spatial memory was assessed using Ethovision XT tracking software to record number of entries into the novel arm as well as total duration in the novel arm. Total distance moved was also recorded to control for any influence of altered activity.

### Immuno-histochemical Analysis for Amyloid Beta Plaque and Immune Biomarkers

Following the final bleed, mice were transcardially perfused with 10 ml phosphate-buffered saline at 37°C (PBS; containing 50 mM Na_2_HPO_4_, 50 mM NaH_2_PO_4_, 154 mM NaCl; pH 7.4) followed by 50 ml of ice-cold PBS containing 4% paraformaldehyde (PFA; Merck, Hohenbrunn, Germany). Brains were then removed, hemisected and stored in 10% neutral buffered formalin prior to paraffin embedding.

Immuno-histochemical (IHC) processing was performed by Histology Core Service Laboratory (Florey Institute of Neuroscience and Mental Health, University of Melbourne, Victoria, Australia). Brains sections at 5 µm thickness were assessed for amyloid beta plaque load using 1E8 antibody (mouse monoclonal specific to Aβ17-22, University of Melbourne, Victoria, Australia [104]) with secondary antibody and streptavidin-biotin-diaminobenzidine image development. Image capture was conducted using a Leica DM LB2 microscope (Leica Microsystems Inc. Bannockburn, IL, USA) under 20-fold magnification, in transmittance mode. Amyloid beta peptide plaque loadings were determined by Otsu image thresholding after subtraction of background and interferences from nuclei. Image quantitation and statistical analysis were conducted using Matlab (Natick, MA, USA).

Immuno-histochemical analysis for interleukin (IL)-10, IL-1β and glial fibrillary acid protein (GFAP) were conducted using sections (5 µm) cut of paraffin-embedded formalin-fixed whole brain tissue blocks. Peroxidase IHC was conducted using antibodies against IL-10 (rabbit polyclonal, 2 µg/ml, Abbiotec, San Diego, CA, USA), IL-1β (rabbit polyclonal, 5 µg/ml, Abbiotec, San Diego, CA, USA) and GFAP (goat polyclonal, 5.8 µg/ml, Dako, Golstrup, Denmark), all prepared in 100 mM Tris buffer, pH 7.4. For IL-10 and GFAP, antigen retrieval was conducted by microwave heating for 15 min in citrate buffer (0.2 M sodium citrate, pH 6.0), prior to applying antibodies to IL-10 and GFAP. Sections were treated with proteinase K (undiluted, Dako) for 4 min at 22°C prior to treating with the antibody to IL-1β. Brain sections were then treated with 1% peroxide in 50% ethanol for 30 min to inhibit endogenous peroxidase activity before blocking in 10% (v/v) serum (Invitrogen, Carlsbad, CA, USA) in 100 mM Tris buffer, pH 7.4. Brain sections were incubated with primary antibodies (1 h at 37°C) and rinsed (3×5 min) before adding biotinylated anti-mouse/rabbit secondary antibody (Vector Laboratories, Burlingame, CA, USA, 30 min at 37°C) followed by streptavidin-conjugated horseradish peroxidise (Vector Elite ABC, Vector Laboratories, 30 min at 25°C) and then with 3,3′-diaminobenzidine (DAB) in 3 mM H_2_O_2_ (20 min at 22°C). Sections were lightly counterstained with cresyl violet (0.5% w/v, aqueous solution) before rapid (30 s) dehydration with ethanol washing (1×70%, 1×95% and 2×100%), followed by xylene washing (2×100%) and coverslipped with Distrene Polystrene Xylene (DPX, Sigma). Negative controls were prepared by omitting the primary antibody however no staining was observed in negative control sections.

Stained sections were blind-evaluated by brightfield microscopy (Olympus BX31 camera, Olympus, North Ryde, Australia) with quantitation performed using a 10×10 eyepiece graticule. The IL-10 and IL-1β-positive neurons were quantified in 4 graticule fields measuring 500×500 µm at 400-fold magnification. Graticule fields were chosen randomly, avoiding any tissue areas damaged during processing. Standard inclusion (upper and right) and exclusion (lower and left) borders were used. Neurons were evaluated on the basis of morphological features including size, amount of cytoplasm and presence of a nucleolus. Automated image analysis was conducted using Java-based open source image processing software (ImageJ v1.44, Bethseda, Maryland, USA). Briefly, images were converted to binary after applying a colour threshold, the number of particles was then analysed either as a ratio or according to clustering and sphericity for standardised areas.

### Statistical Analysis

Four-way repeated measures ANOVA testing was performed using SPSS (Version 16, Quarry Bay, HK). All other statistical analyses were performed using SigmaStat (Version 3.5, Aspire Software International, Ashburn, VA, USA). For all tests, significance was at *p*<0.05, unless otherwise stated.
